# Development of a physical literacy model for older adults – a consensus process by the collaborative working group on physical literacy for older Canadians

**DOI:** 10.1186/s12877-017-0687-x

**Published:** 2018-01-16

**Authors:** Gareth R. Jones, Liza Stathokostas, Bradley W. Young, Andrew V. Wister, Shirley Chau, Patricia Clark, Mary Duggan, Drew Mitchell, Peter Nordland

**Affiliations:** 1 School of Health and Exercise Sciences, Faculty of Health and Social Development, University of British Columbia Okanagan Campus, Kelowna, BC V1V 1V7 Canada; 2 School of Kinesiology, Faculty of Health Sciences, University of Western Ontario, London, ON N6A 3K7 Canada; 3 School of Human Kinetics, Faculty of Health Sciences, University of Ottawa, Ottawa, ON K1N 6N5 Canada; 4 Gerontology Department, Simon Fraser University, Vancouver, BC V6B 5K3 Canada; 5 School of Social Work, Faculty of Health & Social Development, University of British ColumbiaOkanagan Campus, Kelowna, BC V1V 1V7 Canada; 6 Active Aging Canada, Shelburne, ON L9V 398 Canada; 7 Canadian Society for Exercise Physiology, Ottawa, ON K1R 6Y6 Canada; 8 Physical Literacy, Sport for Life, Port Moody, BC V3H 4W6 Canada; 9 Canadian Senior Games Association, Edmonton, AB T6H 4J8 Canada

**Keywords:** Physical activity, Aging, Physical literacy, Mixed-methods, Delphi survey

## Abstract

**Background:**

Arguably the uptake and usability of the physical activity (PA) guidelines for older adults has not been effective with only 12% of this population meeting the minimum guidelines to maintain health. Health promoters must consider innovative ways to increase PA adoption and long-term sustainability. Physical literacy (PL) is emerging as a promising strategy to increase lifelong PA participation in younger age-groups, yet there is relatively little evidence of PL being used to support older adults in achieving the PA guidelines.

**Methods:**

An iterative and mixed-methods consensus development process was utilized over a series of six informed processes and meetings to develop a model of physical literacy for adults aged 65 years and older.

**Results:**

A multi-disciplinary collaborative working group (*n* = 9) from diverse practice settings across Canada, and representative and reflective of the full range of key elements of PL, was assembled. Three consensus meetings and two Delphi surveys, using an international cohort of 65 expert researchers, practitioners, non-government organizations and older adults, was conducted. 45% responded on the first round and consensus was achieved; however, we elected to run a second survey to support our results. With 79% response rate, there was consensus to support the new PL model for older adults.

**Conclusion:**

Older adults are a unique group who have yet to be exposed to PL as a means to promote long-term PA participation. This new PL model uses an ecological approach to integrate PL into the lifestyles of most older adults. Understanding the interactions between components and elements that facilitate PL will ultimately provide a new and effective tool to target PA promotion and adherence for all older Canadians.

## Background

There currently exists a physical inactivity crisis among older adults who are the most inactive segment of the Canadian population [1]. Although the health benefits of physical activity (PA) for an aging population are well established [2–4], the majority of older adults do not accumulate enough PA to receive some level of protection from chronic disease and disability [5]. In no other age-segment of the population is the role of PA for promotion of health and physical independence more applicable and crucial than for older adults. The first wave of the Baby Boomer cohort reached 65 years of age in 2011, and coupled with increasing life expectancy, one in four Canadians will be an older adult over the next 20 years. This population wave of older adults is already being experienced throughout most European countries; where PA levels vary depending upon factors such as income and the availability of formalized social support networks [6]. Physical activity guidelines have been developed to provide older adults with valuable information on what they must do in order to maintain and/or improve health. However, the uptake and usability of these guidelines, globally, have not yet to any large degree been effective in increasing PA participation by older adults, which should be of concern to health promotion specialists [7]. Therefore, addressing a change in PA levels is critical if policy makers and health promoters are going to effectively influence the PA behaviours of older Canadians and older adults across the globe.

The promotion of physical literacy (PL) is emerging as a promising strategy to increase lifelong PA participation in younger age-groups of the population [8]; however, there is relatively little evidence of PL being used to support older adults in achieving the PA guidelines. Physical literacy is defined as “*the motivation, confidence, physical competence, knowledge and understanding to value and take responsibility for engagement in physical activities for life”* [8]*.* Physical literacy is highlighted as the basis of the Canadian Sport for Life Long-term Athlete Development Model [9] which seeks to increase the involvement and enjoyment of sport for all Canadians. In the older adult population, the ability and confidence of an individual to participate in various physical activities is a strong predictor of life-long participation in healthy sustaining PA opportunities [10, 11]. This is of great importance as recent national surveys suggest that only 12% of older Canadians (60–79 years) actually achieve minimum levels of PA required to maintain health [12]. Increased participation in PA and the subsequent maintenance of physiological function can help to alleviate negative attitudes towards the aging process [13, 14]. In addition, having PA opportunities that match the older adult’s physiological capacity will also help to reduce self-reported barriers to PA participation [12]. These components are already included within existing PL models; developed for athlete development and for children and youth only [9, 15]. Thus, we proposed that a PL model be developed and used for older adults to sustain lifelong PA participation and as a strategy for PA advocates, promoters and facilitators to support PA guidelines. Physical literacy could be the elusive factor that will make a successful and sustained increase in PA participation by older adults [16].

The literature is most established in describing PL in children and youth. However, these models of PL for children and youth and the Canadian long-term athlete development model may not be appropriate when applied to older adult populations [17], as they are rooted in development of PL from childhood and do not consider topics of retention, loss and re-tuning of skills at life stages by which individuals negotiate age-associated physiological decline. The current Canadian long-term athlete development model highlights the importance of PA skill development and the use of these skills within various environments. Within an inactive older adult population, the primary interest may not be kicking or throwing skills, but rather the re-training of functional skills that will assist in maintaining physical independence and preventing frailty [18]. There is a dearth of research that has explored PL in older adults. A search of pertinent literature databases by our librarian using the relevant keywords ‘physical literacy’ and ‘aging’, yielded no evidence-based citations that directly describe the concept of PL in the older adult population. Consequently, there currently does not exist an approach to frame PL for older adults.

There have been parallel developments in the area of health literacy, defined as “the degree to which individuals have the capacity to obtain, process, and understand basic health information and services needed to make appropriate health decisions” [19]. Applied to older adults, lifelong educational and learning practices (self-study, computer/Internet and print resources, etc.) have been modelled as important enablers for positive health behaviours [20]. Such pedagogical development emphasizes the importance of generic health literacy resources and highlights the methods used by older adults to access and learn positive health behaviours that foster optimal aging. Similarly, a PL model extends this health literacy work by uniquely specifying evidence-based PA behaviours and practices for all older adults across the latter stages of their life course.

The purpose of this manuscript is to describe the process used to develop a model of PL in the context of the older adult population. Ideally, the model will represent specific identified components that support PL in the older adult because the role of PL in later life PA participation is currently unknown. Due to low PA levels, attitudinal, societal, cultural, gender, and environmental factors, it is hypothesized that the current older adult population likely possess little knowledge of or engagement with PL; contributing to their lack of PA participation. Thus, the specific objectives of this project are to: (1) Assemble a collaborative working group of researchers and stakeholders whose expertise and reach cover a broad perspective of PA and aging; and (2) Develop an evidence-based model of PL for older adults. The aim of this project is to develop a framework that promisingly captures integral aspects of PL that validly organizes and presents key facts in a manner that can be used to guide informational approaches that promote PL with respect to knowledge exchange among older adults, knowledge use by practitioners, and knowledge creation by researchers. With this aim of moving towards a seminal model, we anticipate that a refined model could, in the long run, be employed in efforts to increase awareness and provide education in the area of PL. As such, this model would eventually become a meaningful resource for increasing PA to appropriate levels and reducing the sedentary behaviours of older Canadians and eventually older adults across the globe.

## Methods

A collaborative working group (CWG) of expert researchers and knowledge users (2 were older adults 65+ years) were identified and assembled to undertake this initiative. This CWG was a multi-disciplinary team from diverse practice settings across Canada and representative and reflective of the full range of key elements of PL including affective, physical, cognitive and behavioural factors as outlined in the 2015 Canada’s Physical Literacy Consensus Statement. Specifically, the CWG consisted of individuals with expertise in; exercise physiology and aging (GJ, LS), psychosocial and socio-cultural aspects of exercise and aging (SC, AW, BY), professional development and promotion in the area of exercise (MD and GJ), sport pedagogy and older sportsmanship (BY), knowledge translation in the area of active aging (PC, GJ, and LS), older adult sport organization (PN), physical literacy (DM), and gerontology and policy (AW). All members of the CWG were involved in each step of the consensus development process. The number of CWG members (*n* = 9) was based on 9-member RAND panels [21]; large enough to permit diversity of representation while still small enough to allow everyone to be involved in the group discussion.

**Fig. 1:**
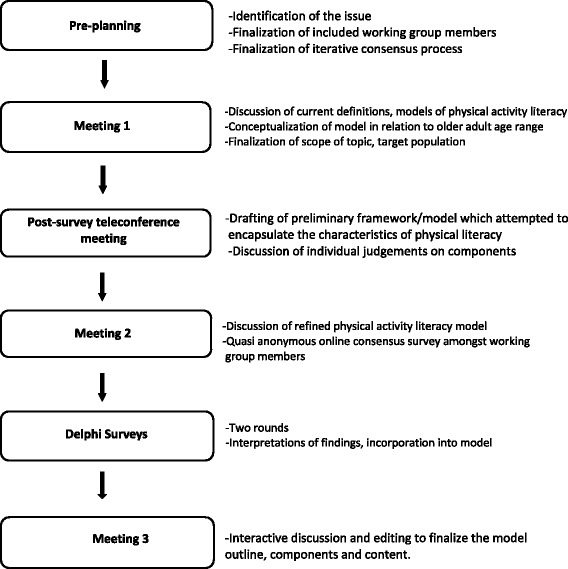
Consensus Development Process

An iterative and mixed-methods consensus development process was utilized (Fig. 1), over a series of six informed processes and meetings. This method was used to collate and consider the best available evidence using the collective judgement of these experts to yield a consensus for the purpose of developing the model. This study received ethical approval from the University of British Columbia’s Behavioral Research Ethics Board [Ref# H17–00884] and was in compliance with the Helsinki Declaration. All interview participants gave consent to participate in the survey via email response.

### Pre-planning

In advance of our preliminary meetings, a systematic review of the literature was completed to identify, appraise, and synthesize studies related to PL literature related to older adults [21]. The results of this review revealed that there was a plethora of research available for younger populations, but very limited, if any, research was available on PL in older adults. Development of a PL model for older adults would assist health promotion experts in increasing PA toward evidence-based recommended levels [2, 4].

Pre-planning for the initial formal meeting was conducted in a teleconference format, organized by the working-group leads (GJ and LS). The review of the literature suggested that a PL model for older adults did not exist and therefore the group was introduced to current definitions and other models of PL for children and youth [15, 22]. Discussion focusing on whether these were relevant and appropriate for the older adult population ensued (topic assessment). The CWG agreed that the conceptualization of a potential model in relation to older adult age range was warranted, and therefore pre-planning proceeded with the discussion of topic refinement. The CWG adopted the operational definition of older adults to include men and women, aged 65 years and older, living independently in the community. The CWG acknowledged the diversity of the older adult population and indicated that future refinement of a PL model would need to be inclusive of sex, gender, ethnicity and socioeconomic status. The CWG also proposed that the model would comprise a framework to better understand the competencies/modalities to assist this population to meet the current PA guidelines for older adults. To that end, the CWG agreed to use the current Sport for Life PL model [23] as a guide. However, because of issues of entry points, baseline PL levels, and potential re-entry points (varying across the life course), the CWG decided to use the International Physical Literacy Association’s definition of PL; “*the motivation, confidence, physical competence, knowledge and understanding to value and take responsibility for engagement in physical activities for life”* [8]. The CWG leads were tasked with developing a preliminary framework to act as a starting point for meeting one.

### Meeting one

**Fig. 2:**
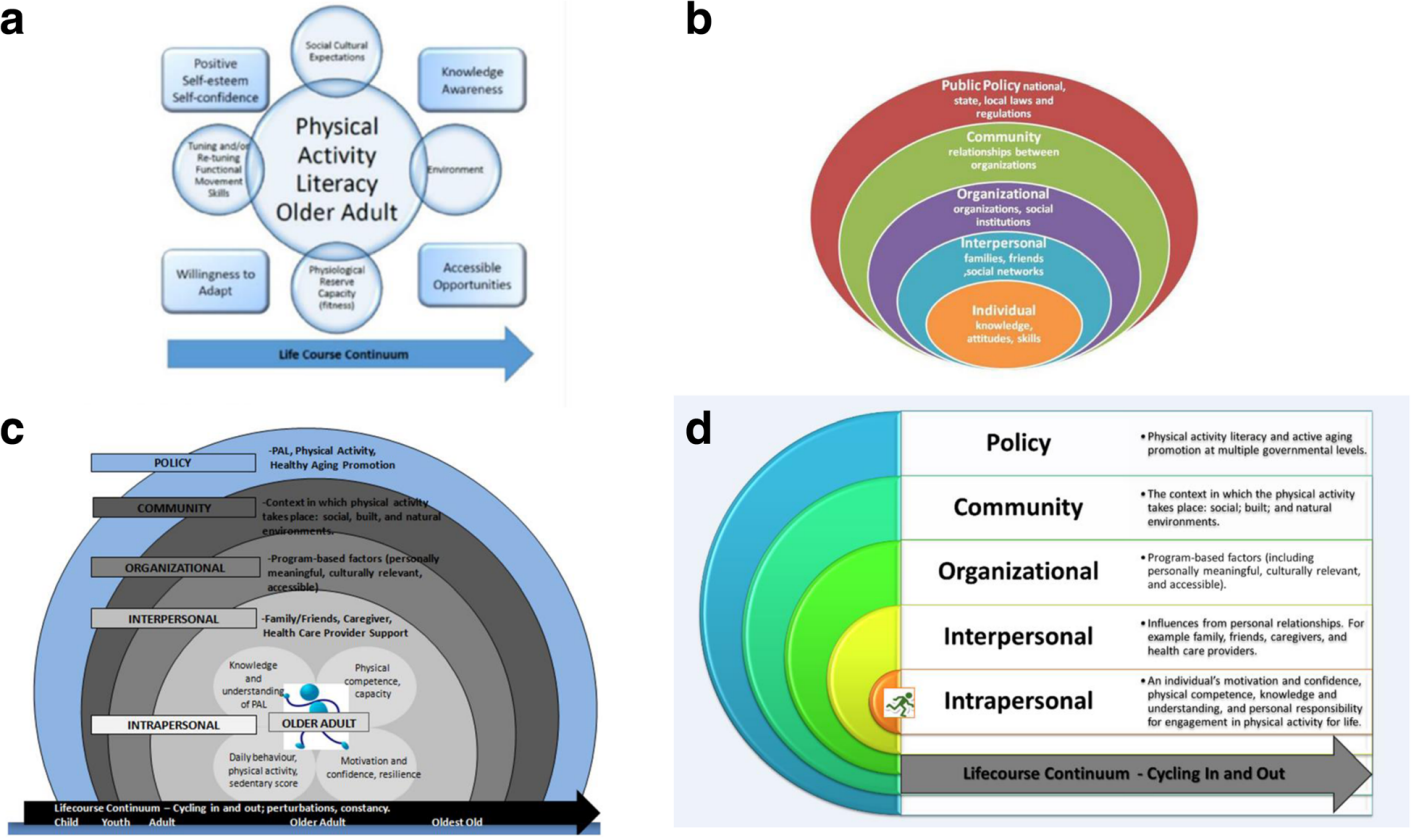
Evolution of the Physical Literacy Model for Older Adults

Meeting one was conducted using a teleconference format. The CWG leads presented a preliminary framework of PL in older adults that was distributed ahead of the meeting (Fig. 2a). The preliminary framework attempted to encapsulate the characteristics central to PL, supported by factors that influence these characterisitics within the context of older adults and the CWG’s expert opinion. The components essentially represented an indication matrix related to PL for older adults. Using a nominal group technique, each of the components was discussed as to their relevance, importance, and influence on PL by the expert CWG. Overall, the discussion of the components emerged to be heavily framed within a broader definition of PL, ultimately leading to a consensus that, moving forward, the model would be anchored by componenets at the individual level (representing a core set of physical skills and physiological and psychological capacities) and would further evolve into an ecological model, which considered various other broader aspects of the surrounding social and physical environment, and constituent organizations (programs and services) that come to bear on older adults and how they might optimize their PA participation.

In order to populate this preliminary list of indications, the CWG was tasked with providing extended feedback following meeting one. Specifically, each CWG member was asked to provide, via email, individual judgements on components (socio-cultural expectations, environment, physiological reserve capacity/fitness) and related influencing factors (tuning and/or retuning functional movement skills, knowledge and awareness, accessible opportunities, willingness to adapt, positive self-esteem and self-confidence). In addition, feedback was requested with respect to the context of components, how each component might be assessed, and how each might be maximized to induce changes in PA levels. Feedback was summarized by the CWG leads and incorporated in the next draft of the model, discussed at Meeting two. Literature searches to confirm evidence-basis and best-practices for included components and influencing factors (as they relate to the older adult population) were conducted [24].

### Meeting two

Meeting two was conducted via teleconference. The CWG leads presented a refined framework on PL in older adults, distributed in advance of the meeting (Fig. 2b).

Refinement of the framework resulted in an expansion and restructuring of the model to reflect a socio-ecological framework. Thereafter, there were three steps to further refine the model: 1) An assessment of Canada’s Physical Literacy Consensus Statement; and 2) Consensus that the four elements outlined in Canada’s Physical Literacy Consensus Statement translate to an older population; and 3) Specifying the statement to the older adult. As such, each component of our emerging model was harmonized with identified elements from Canada’s Physical Literacy Consensus Statement. The CWG acknowledged that the four elements outlined within the statement would support older adults, at least at the individual level, on how to succeed with lifelong PL and were described as:

1. **Knowledge and understanding** about successful aging, including what physical, psychological and social determinants influence well-being and what past experiences might help support or impede PL;
2. **Physical competence and capacity**, acknowledges that older adult maintain physical capacity (fitness) in order to be able to engage in PA and sport. Older adults can be educated on the consequences of age-associated physiological decline and how that impacts PL;
3. **Motivation and confidence** will foster resilience to age-related decline and the accumulation of comorbidity throughout the aging process (allowing older adults to adapt); and
4. **Responsibility and understanding** of current PA behaviours (i.e. steps per day, min/week, and reducing sedentary behaviours) so that an appropriate dose of PA may be achieved that promotes health, fitness and disease prevention.

Individual panel members were provided an opportunity to make full and equal contribution through a post-meeting quasi-anonymous online consensus survey. The survey was quasi-anonymous because, although the CWG were aware that all members were participating; the online responses had no identification tags, making the responses quasi- anonymous. In addition, the CWG completed a knowledge resource nomination worksheet, which assembled and categorized additional content experts and stakeholders in the broader field of PL (e.g. coaches, physical educators, etc.). The CWG leads summarized the results of the panel consensus survey and integrated them into the model (Fig. 2c) that would be used within our Delphi survey.

### Delphi survey

**Fig. 3:**
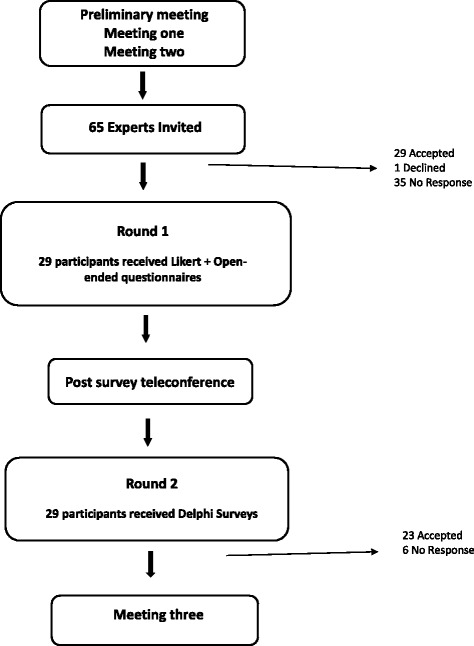
Delphi Survey Process

In order to increase the rigor and confidence of the developed framework and to obtain broader consensus, a Delphi survey was conducted. As McKenna [25] has noted, the Delphi technique is most useful when the research objective is to correlate informed judgements on a topic spanning a wide range of disciplines, as is the case in this initiative. The Delphi technique involved an iterative, multistage process by which multiple rounds of questionnaire data collection were conducted (Fig. 3). A web-based survey served as the mechanism for administering the questionnaires. To reach consensus, agreement was required by 75% of responders. This level of agreement determined the number of rounds used to administer the questionnaire. Significant consensus was achieved in round one for each of the elements of our PL model (i.e. percentage responding either somewhat agree, agree or strongly agree); however, an additional round with modifications was circulated to garner consensus related to editorial modifications to the model. These modifications were approved during a post-survey teleconference meeting with the CWG.

### Post-survey teleconference meeting

The CWG conducted a teleconference meeting to discuss the first round of the Delphi survey. Group members provided feedback on the ratings, focusing on areas of disagreement or suggestions provided. A revised model was distributed to the 29 respondents who completed the first round. Again, Delphi survey expert participants reached significant consensus that the proposed model was representative of the concept of PL for an older adult population. Feedback from round two was distributed to the CWG, via email, ahead of Meeting three.

### Meeting three

Meeting three was conducted in-person in Toronto, Ontario in September 2016, with seven out of the 9 CWG members participating and the remaining providing feedback via email. Meeting three began with a review of the process to date. Then a review and discussion of feedback from participants of the Delphi survey second round was conducted and consensus was reached on edits to the PL model by the Delphi process. Following this, an interactive discussion and editing of the model took place, with the goal of finalizing the model outline, components and content.

## Results

### Delphi survey

The Delphi survey was sent out to 65 international experts in PA and aging. This included researchers, practitioners, non-governmental organizations and older adults engaged in physical activity promotion. Twenty-nine (45%) completed the survey, three were older adults (65+ years) (Table 1). The distribution of all the Delphi participants’ first round ratings are presented in Table 2. Twenty-three (79%) out of the original 29 respondents completed the second round of the Delphi survey. Of those, 17 provided their names to be included in the present manuscript. Results of the second round Delphi survey are presented in Table 3.

**Table 1:** Round 1 Delphi Survey Participant Characteristics

Characteristic	Frequency
Sex (26/29)	
Female	17
Male	9
Country (27/29)	
Australia	3
Canada	16
Italy	1
Japan	1
United Kingdom	3
United States of America	3
Occupation (26/29)	
Researcher	17
Educator	2
Medicine	1
Non-profit Volunteer with National Organization	3
Professor	1
Professor and Masters Athlete Coach	1
Kinesiologist	1
Occupation involves working specifically with older adults (27/29)	
Yes	20
Older adults are one sub-population	5
Indirectly, research	2
Number of years in this occupation (25/29)	
0–10 years	7
11–20 years	2
21+ years	18
Area of Expertise (26/29)	
Exercise physiology (neuromuscular, musculoskeletal, metabolic)	5
Falls prevention / Injury prevention and aging	4
Physical activity and aging	3
Chronic conditions and aging	2
Policy / advocacy	2
Social theory and sport/physical activity participation	1
Gerokinesiology	1
Geriatrics	1
Coaching	1
Exercise and cognition	1
Mobility and aging	1
Successful Aging	1
Physical culture of the aging body	1
Housing, health promotion, elder abuse, disasters, gerontechnology	1

Responses to the specific question/responses to the questionnaire

**Table 2:** Round 1 Delphi

Question Posed	Strongly Agree	Somewhat Agree	Agree	Neutral	Disagree	Somewhat Disagree	Strongly Disagree	Cannot Adequately Respond
Q1. An appropriate way to frame the intrapersonal or ‘individual’ level factors associated with physical activity literacy in older adults is via the ‘elements’ of physical literacy: motivation and confidence; physical competence; knowledge and understandings; and engagement in physical activities for life. (28/29)	11 (39%)	5 (18%)	7 (25%)	2 (7%)	1 (4%)	1 (4%)	1 (4%)	0
Q2. The “Interpersonal” level factors of the model are appropriately described by family, friend, caregiver, and health care provider influences. (27/29)	12 (44%)	7 (26%)	5 (19%)	0	2 (7%)	1 (4%)	0	0
Q3. ‘Organizational’ level factors are appropriately described by program-based factors that offer personally meaningful, culturally relevant, and accessible physical activity opportunities. (26/29)	10 (39%)	6 (23%)	8 (31%)	0	2 (8%)	0	0	0
Q4. It is appropriate to frame ‘Community’ levels factors in the context in which the physical activity takes place. This includes the social, built, and natural environments. (27/29)	17 (63%)	3 (11%)	6 (22%)	0	1 (4%)	0	0	0
Q5. At the ‘Policy’ level, it is appropriate to include physical activity literacy, physical activity or healthy aging promotion initiatives across various levels of government. (27/29)	14 (52%)	4 (15%)	7 (26%)	1 (4%)	1 (4%)	0	0	0
Q6. The International Physical Literacy Association’s definition of physical literacy (below) is appropriate for the older adult age range. “Physical literacy is the motivation, confidence, physical competence, knowledge and understanding to value and take responsibility for engagement in physical activities for life.” – International Physical Literacy Association, May, 2014. (27/29)	9 (33%)	6 (22%)	6 (22%)	2 (7%)	3 (11%)	0	1 (4%)	0
Q7. Overall, the proposed model is an appropriate way to visualize physical activity literacy in older adults. (27/29)	10 (37%)	7 (26%)	8 (30%)	0	2 (7%)	0	0	0

Responses to the specific question/responses to the questionnaire

**Table 3:** Round 2 Questions and Level of Agreement

Question Posed	Strongly Agree	Somewhat Agree	Agree	Neutral	Disagree	Somewhat Disagree	Strongly Disagree	Cannot Adequately Respond
Q1. An appropriate way to frame the intrapersonal or ‘individual’ level factors associated with physical activity literacy in older adults is via the ‘elements’ of physical literacy: motivation and confidence; physical competence; knowledge and understandings; and engagement in physical activities for life. (23/23)	14 (61%)	3 (13%)	4 (17%)	2 (9%)	0	0	0	0
Q2. “Interpersonal” factors of the model are appropriately described by a spectrum of formal and informal personal relationships. (23/23)	14 (61%)	6 (26%)	2 (9%)	1 (4%)	0	0	0	0
Q3. ‘Organizational’ factors are appropriately described by evidence-based physical activity programs and services and physical activity opportunities that offer personally meaningful, culturally relevant, and accessible physical activity opportunities. (23/23)	16 (70%)	1 (4%)	2 (9%)	3 (13%)	1 (4%)	0	0	0
Q4. ‘Community’ encompasses the context in which the physical activity takes place: includes social connectedness and social-capital building; socio-cultural norms and expectations; and affordances for physical activity within the built and natural environments. (23/23)	12 (52%)	4 (17%)	5 (22%)	0	1 (4%)	0	0	1 (4%)
Q5. ‘Policy’ factors include physical activity literacy, physical activity or healthy aging promotion initiatives across pan-governmental and multi-sectorial levels and including non-governmental organizations. (23/23)	13 (57%)	3 (13%)	5 (22%)	1 (4%)	0	0	0	1 (4%)

Responses to the specific question/responses to the questionnaire

### Proposed physical literacy model for older adults

**Fig. 4:**
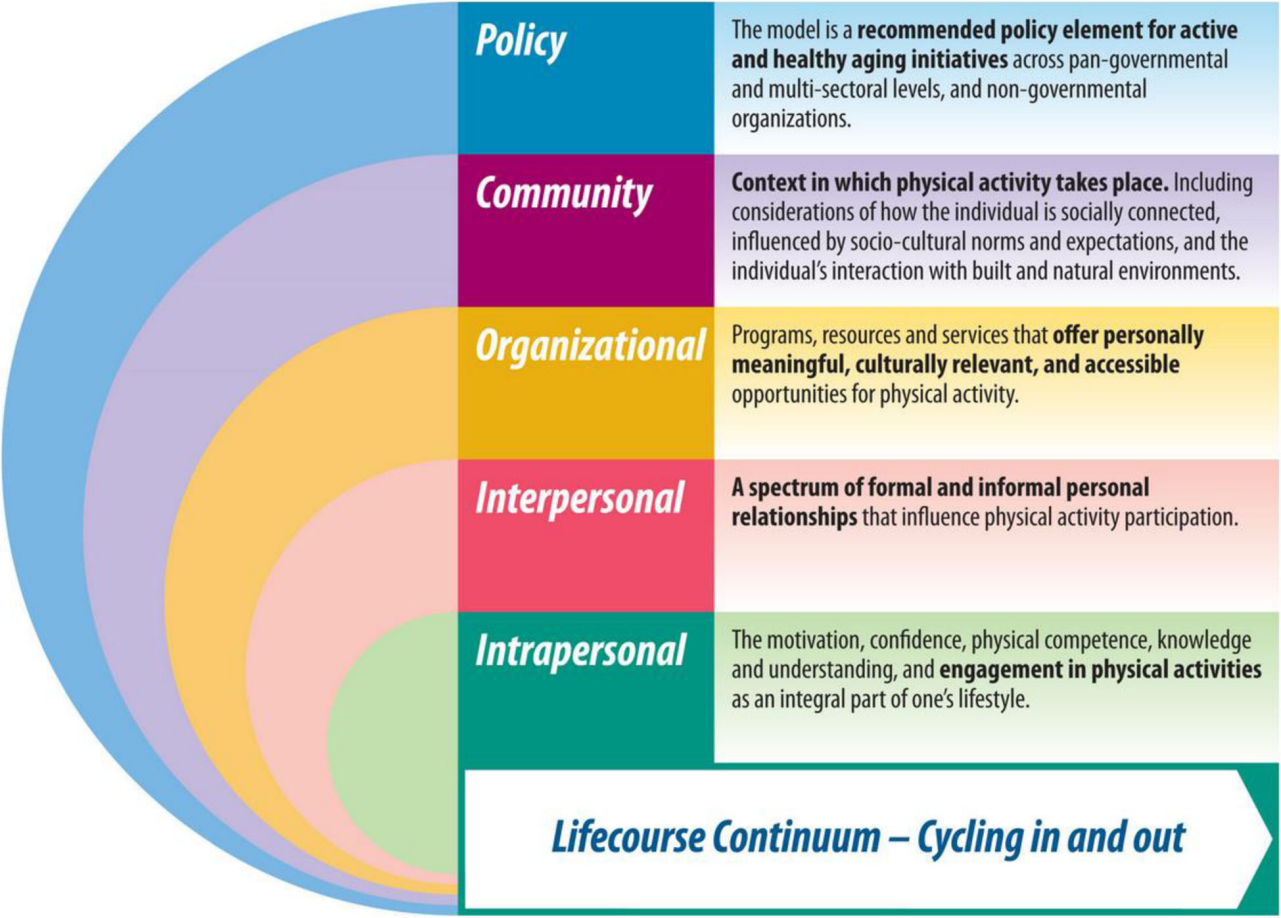
Physical Literacy Model for Older Adults: An Ecological Approach

The final proposed PL model (Fig. 4) is structured with the defining characteristics of core PL competencies (individual/intrapersonal elements) at its core, with an additional four domains (interpersonal, organizational, community, and policy) that may influence both PA participation and the quality of the PA experience. Each domain provides an example of how PL may be utilized to promote lifelong PA in older adults. In particular, the core competencies of PL at the intrapersonal level may be realized to varying degrees, optimized or constrained, depending on conditions in the surrounding domains of this ecological model. Descriptions are provided for each domain to demonstrate the various facts and interactive components that need to be considered in light of PL and the promotion of lifelong PA in older adults.

## Discussion

### Intrapersonal

The individual older adult is at the centre of the PL model. Intrapersonal elements include personal factors reflecting the four elements of the definition of PL, each of which may increase or decrease the likelihood of an older adult becoming or remaining physically active. Strategies that bring change at the individual level focus on an individual’s motivation and confidence, physical competence, knowledge and understanding, and assist in engagement in PA participation as an integral part of one’s lifestyle.

**Motivation** to be physically active in the older adult population can vary from younger populations; primarily influenced by health concerns and anticipated benefits [26–28]. For example, as people age, motives that indicate pragmatic or instrumental concerns, seem to override ones that might be more personally uplifting. This has previously been reported by Trujillo et al. [29] who demonstrated that, as opposed to younger adults who exhibit greater concern for interpersonal attraction outcomes, older adults exhibit greater concern for health outcomes. As such, health and maintaining physical and mental independence may be potent motivators for PA participation in older adults. Although there may be general age-related changes in participatory motives, evidence suggests that motivation to engage in various types of physical activities is multifaceted and draws on a wide range of reasons beyond health and fitness benefits, in both exercise and sport domains. For example, characterises of adaptive motivation may relate to determination (fulfilling needs for autonomy, competency, and relatedness) and whether the motives are personally meaningful and integrated to important values and beliefs held by middle-aged and older adults [30–33].

Adherence to structured exercise programs is consistently associated with higher exercise-related self-efficacy, that is, **confidence** in both performing specific exercises and in planning to exercise [11, 34]. Further, confidence to make and sustain feasible changes and confidence to overcome barriers, are key factors in the likelihood of making lifestyle change among older adults [10]. In addition, as in younger populations, confidence related to current PA participation is shaped by past experiences [35, 36]. Therefore, it is important to gain insight into an older adult’s past PA history; including understanding which PA skills they learned and the context in which they were learned, which skills they may be re-learning, or skills confronted for the first time. Finally, previous adverse events and perceived risks associated with PA participation may also impact confidence. As such, fear of falling or fear of exacerbating health conditions during physical activities are barriers that can be mitigated, for example, through improving balance confidence [37].

The **physical competence** element of PL refers to an individual’s ability to develop and/or re-learn important functional movement skills and patterns, and the capacity to experience these skills through a variety of movement intensities and durations. Current PA models describe a pathway from birth to adulthood and therefore may not apply to older adults who may not have developed any or certain skills (base functional movement skills) or who have not engaged in activities using these skills for many years. Further, the current models reflect a time of growth and development during skills acquisition and again, may not be applicable to the older adult living with age-related physiological changes, who may be more focused on retention rather than regaining past skills or learning new ones. Therefore, an important question toward increasing the physical competence element of PL in older adults includes what changes to the nervous system, motor systems, and motor skill learning will influence ability to engage in acquired movement skills and/or to learn new movements, in light of primary age-related changes of physiological systems?

Age-related declines in physical fitness and performance are such that physical limitations may impinge on functional activities of daily living [2], resulting in higher rates of disability [38], and are associated with all-cause mortality and premature death [39]. It is not surprising that mobility troubles, fear of falling, and health conditions are reported barriers to PA participation among Canadian older adults [40]. Given the episodic nature of many chronic conditions, there may be more treatable moments or thresholds at which time perceived barriers are more, or less, debilitating than at other times. Appropriate exercise training can minimize declines and maximize physical competence, thus mitigating the rates at which older adults cross thresholds of functional inability. In addition, increased participation and competence in PA can reduce negative attitudes towards the aging process [12–14]. This is analogous to applications of the concept of resilience to coping with illness among older adults, broadly defined as a dynamic adaptive process through which individual traits, characteristics of their environment, and their internal and external resources, and physical capacity, are utilized in the face of adversity [41]. Older adults are capable of resilience to adverse health events despite socioeconomic backgrounds, personal experiences, and declining health. Research suggests that strong mental, social, and physical characteristics are associated with better resilience among older adults [42]. Physical activity and social engagement often associated with functional resilience are considered fundamental in coping with chronic disease and multimorbidity, which are common in older age groups [43].

An older adult’s awareness and comprehension of the PA guidelines, understanding of the role of PA in healthy aging, knowing about movement skill parameters, methods of improvement, and safe participation modifications are all **knowledge and understanding** elements of PL. Similar to younger populations, older adults tend to have limited knowledge of current PA recommendations for their age-group [44] and on accruing appropriate intensities for meaningful health benefits [45]. Physical activity interventions that include an educational component addressing these elements can increase outcome expectations, skills knowledge, and knowledge on effective doses and types of PA [46]. In addition, older adults should have knowledge and understanding of what barriers to PA and sport participation exist. Not enough time, lack of motivation, ageism and feelings of being too old, perceiving few sport facilities and/or physical activity opportunities nearby, and lack of support from others, are all recognized as consistent barriers for older adults. The literature on perceived barriers to participate in PA and sport suggests that these challenges are consistently reported among older adults [34, 47]. These barriers are all influenced by how older adults view themselves and how they are cognizant of, and understand the ecology and opportunities surrounding them.

There is vast room for improvement in encouraging older adults to make the choice to be physically active. Along with previously identified motivators and barriers, **prioritizing and sustaining engagement** in physical activities as an integral part of one’s lifestyle can be influenced by outcomes expectations, perceptions of older age and attitudes towards aging and exercise. The belief that a PA behaviour, in this case PA, will bring about a certain consequence (outcomes expectations) and identifying which sub-category (physical, social and/or self-evaluative) is personally meaningful may further increase engagement [48]. Negative stereotyping of old age (including cultural, societal stereotypes) and low expectations for old age, may interfere with the possibility for improvement via healthy lifestyle behaviors [28]. For example, a sample of inactive older persons perceived themselves to be physically active, because their perception of PA was grounded in a social context [27]. Both of these perceptions may interfere with the recognition and value of regular PA as a personally meaningful and integral part of life. Conversely, highly active older adults utilize their resourcefulness to support their PA and in turn, PA contributes to their definition of self [48]. Similarly, literature on adult sport [49] [30, 50], indicates that negative attitudes and feeling too old to engage in sport are common barriers constraining activity. This behavioural PL element suggests a role in assisting older adults to link the value of, or belief in PA and behaviour change to regular PA participation.

Finally, at the intrapersonal level, there are individual factors identified to be unique to the PA levels of older adults. For example, differences between males and females or variations across the older adult age-range. Other groups at risk for low PA levels include, women, older adults with low incomes and/or low education levels, older adults living with disabilities and/or chronic health conditions, those who live in institutions or in isolation, and seniors who are members of ethno-cultural and ethnolinguistic minority population groups [51]. Each individual has a cultural identity and understanding cultural context can act as starting point to assist older adults. In addition, there must also be consideration for examination of PL from a life course perspective. Such a broad perspective is important for all of these identified factors, which requires a flexible and tailored approach to PL. Although children and youth are likely to have some continuity to participation in sport and PA, older adults are more likely to cycle in and out of the model as they advance across the lifespan. This ebb and flow pattern of PA is likely to be partially driven by intrapersonal engagement, and how many of the identified individual factors interact with social opportunities, seen at the interpersonal, organizational and community levels of the model.

### Interpersonal

Interpersonal elements that influence PL in older adults are described by a spectrum of formal and informal personal relationships, often broadly termed social support. **Personal relationships** such as family, friends, and broader personal social networks such as work/volunteer peers, caregivers, health providers may influence PA participation among older adults [52]. While extensive research on each of these is limited, they represent potential sources (positive and negative) of interpersonal messages and varying types of support influencing older adult’s understanding of PL. A shrinking social circle (especially if they lose an exercise buddy) may negatively influence PA participation with age [53], as may low social support from a ‘significant other’ [54], or from friends [55]. In older adult clinical populations, family support for PA may be lacking out of fear of harm [56]. Conversely, positive personal social support from family, friends, and neighbours can be enablers for PA [57–59] as can be co-participants and PA leaders [60]. Social support through faith-based network positively supports PA participation [61, 62]. Primary care physicians are often identified as having an effective role in counselling older adults on PA. Ultimately, such actions would engender PL in the patient, particularly if it is addressed within the context of a health problem [58, 63, 64]. Understanding the influence personal relationships can have on fostering PL in older adults is of importance, specifically to facilitate individual behavior change. By affecting social and cultural norms and overcoming individual-level barriers to organized programs and services, that support participation in lifelong PA we will be able to facilitate a deeper understanding of PL by the older adult.

### Organizational

**Organizational** elements that influence PL in older adults are described by programs, resources, and services that offer personally meaningful, culturally relevant, and accessible opportunities for PA participation.

With respect to program factors, in September 2007, the National Coalition on Aging, the National Blueprint Office, and Active for Life in the U.S. convened a meeting entitled “Building on Best Practices: Physical Activity Programming in the Aging Network”. This meeting addressed issues related to widely disseminating information on best practices and evidence-based programs to community organizations that serve older adults. The meeting highlighted the importance of selecting evidence-based PA programs to optimize health outcomes, promoting current guidelines, the importance of developing user-friendly resources to increase program access and support, and the importance of quality program evaluation of these initiatives. In addition, Stewart et al., [65] highlighted the need for community physical-activity-promotion programs to be integrated into settings that have the infrastructure, culturally competent staff, access to exercise specialists, and experience in providing outreach and delivering the program to diverse populations. Culturally appropriate interventions have shown mixed results as to their advantage compared to standard interventions; however, most studies are limited due to small target populations, short follow-up, and methodological problems [66].Yet, they signal the importance of expanding frameworks for practice to be consistent with the reality of diverse community contexts and individuals engaging in pluralistic options and hybrid approaches of PA [67].

An important aspect of user-friendly and accessible programming that can influence PL relates to the quality of leaders and coaches associated with PA programs [68]. Curriculum guidelines outlining educational standards for exercise leadership of older adults are available [69]. Nevertheless, in the exercise domain, the quality and relatability of a group leader can be recognized as a factor to motivate and increase older adults’ adherence to PA [60]. Peer-led activities, where older adults are matched with peers also demonstrate increased retention to PA programs [70, 71]. Older adults who participated in a fitness program with peer mentors had improved well-being, improved social functioning, enhanced ability to carry out physical and emotional roles, and increased vitality [72]. In seniors sport, emerging work underscores the importance of coaches who can relate to, and understand, the nuances of interacting with mature older adults [32, 73]. For example, effective leaders often take instructional steps or collaborative conversations to satisfy older adults’ need to know the rationale for why they are practicing something before they undertake it and afford opportunities to self-direct when it is reasonable or safe to do so. Effective adult sport coaches engage in more collaborative conversations and learner-centered questioning during learning activities than they do with younger participants. Not all coaches use such measures, nor do all older adults prefer such approaches (based on given situations and the goals for learning) [74]. However, this work suggests that quality PA experiences depend to an extent on tailoring instructional leadership and programs to older adults’ preferences. Such considerations would plausibly come to bear on intrapersonal factors related to motivation, competency, knowledge, understanding and responsibilities toward PA.

While public health promotion focuses largely on group fitness programming for older adults, there is evidence to support the observation that many older adults prefer to exercise independently (or with some instruction either directly or through media-based programs) rather than in a group setting or class-based setting [11, 75, 76]. Therefore, there is a need to promote a wide range of options [77]. Evidence from trials comparing multiple long-term interventions suggests that mode of delivery is not necessarily important for effectiveness but that tailoring the intervention to participants may be important [78]. It has also been identified that interventions and promotion needs to occur at multiple levels in a variety of settings, and utilizing different technologies and modalities, that fully take into account determinants of PA [75]. Perceived lack of accessibility to nearby facilities due to transportation barriers or functionally appropriate opportunities is also a valid consideration [30, 45, 47, 50]. In addition, there is need for qualified exercise specialists who will be able to administer effective programming to an older adult population with varied needs and abilities [69].

Overall, these strategies are intended to facilitate individual behavior change by influencing organizational systems, leveraging resources and participation of community institutions, and advocacy groups, which represent potential sources of support and communication. Strategies for optimizing programs and building capacity in various organizations facilitate interactive support more broadly at the community level.

### Community

**Community** elements that influence PL in older adults include the context in which PA takes place. This includes considerations of how the individual is; socially connected, influenced by socio-cultural norms and expectations, and their interaction with the built and natural environments where they live.

#### Socio-cultural norms and expectations

There is a growing body of literature on the importance of culturally appropriate PA among ethnic minorities [79]. Cultural predisposition may positively or negatively influence PA participation. For example, some cultures view structured PA as having social meanings tied to oppression embedded in history that may not be in the consciousness of “mainstream” society [80]. Alternatively, faith-based PA and education interventions have had a positive influence upon participation by minority groups [62]. Effective facilitative factors in the context of PA among older adults of cultural diversity include; folk dancing [81, 82] and qi gong [83] or tai chi [84, 85] as well-known examples among the South Asian community. Although, these forms of PA are not usually viewed as such from a Western perspective, they are forms of PA that may be considered extensions of cultural practice and expression. Such forms of PA also promote social support and social inclusion through, group connection and shared understanding of the cultural meaning of the dance and collective movement [86]. Indeed, such forms of PA have become increasingly viewed as complementary and alternative to Western forms of PA and they provide insight into the social connectedness within the community environment that can facilitate greater knowledge surrounding PL.

#### Built environment

The nature of a neighborhood built environment can be an important consideration for older adults’ health and functioning [87] and can determine an older adults’ PA level [88]. Carlson et al., [89] demonstrated that a supportive environment for PA, that has; good walkability, good access to parks and recreation facilities, and good neighborhood aesthetics, was associated with increased moderate-to-vigorous levels of PA in older adults. The existing literature suggests that mobility among older adults in urban areas is associated with higher street connectivity leading to shorter pedestrian distances, street and traffic conditions such as safety measures, and proximity to walkable destinations such as retail establishments, parks, and green spaces [90]. Beyond encouraging walkability, design features can be critical for promoting and maintaining social engagement as well [87]. As such, there is a growing body of literature that supports the study of design features in communities to support mobility for their aging populations. However, a lack of consensus regarding the definitive association between the specific components of the built environment and PA among older adults exists due to various methodologies employed, various settings studied, and the discrepancies between perceived and actual environmental conditions [64]. Specific to the role of the environment in increasing walking in older adults, features such as sidewalk functionality, safety from traffic (including curb cuts), and having proximal destinations are associated with increased walking in older adults [91]. Age-friendly communities address active aging as a key component of their development and such approaches could be better-informed through research elucidating PL processes and interventions [92]. For instance, while promoting walking trails and green spaces for older adults, our model suggests a more coherent multi-level approach that addresses the complex interrelationships at the micro, meso and macro levels affecting PA involvement and ultimately PL engagement.

#### Natural environment

Neighbourhood design that facilitates outdoor walking may be one avenue whereby PA levels of older people can be enhanced, with benefit across socioeconomic strata [93]. In addition, interactions with landscapes embedded with therapeutic qualities including parks, gardens, street greenery, lakes, and ocean views can influence older adult’s perceived physical, mental, and social health. Issues of safety, accessibility, and personal perception have been shown to complicate this relationship [94]. Finally, severe fluctuations in weather (hot summer, cold winter) may influence PA modality variations in older adults [95, 96]. In such cases, being physically literate may help to mitigate the effects of climate on PA participation, through comprehension of alternative PA options.

### Policy

The multidimensional PL model presented in this paper is the product of the expertise and knowledge of a large multidisciplinary team of researchers and stakeholders engaged in PA knowledge translation aimed at increasing PA levels. At the outer-edge of our ecological model resides the **policy** component which is integral to all other components within the model. Policy is what will shape and support elements within the model that facilitate lifelong PA adoption. This model developed specifically for older adults is a recommended policy element for active and healthy aging initiatives across pan-governmental and multi-sectoral levels, and non-governmental organizations. The testing, refinement, and application of a PL model targeting older adults has the potential to be instrumental in improving quality of life, and ultimately the health status, of a rapidly growing older population. To ensure benefits are derived from these approaches, more tools and more effective tools are needed to evaluate, translate, and disseminate research and its findings [97].

Indeed, prevention and maintenance of chronic illness, and enriched quality of life, through the enhancement of PA among older adults has an enormous potential to reduce the burden on the health care system as we move into a period of rapid population aging [97].

The PL model for older adults can be integrated with other major policy developments, such as the age-friendly community movement, national strategies to reduce social isolation [98] and foster community engagement among seniors, ParticipACTION, and prevention components from the National Alzheimer’s Strategy [99], as well as those connected to a National Seniors Strategy [100]. Overall, policy makers must consider all occasions that expose older adults to different movement opportunities and experiences. However, policy makers must also recognise the heterogeneity for both physical and cognitive function observed across the older adult population and as such guide PL programing to be effective at both the marco and micro levels [101].

## Conclusions

Physical literacy is an emerging strategy to remodel how we promote PA participation across the lifespan. Older adults are a unique group who have yet to be exposed to PL as a means to promote long-term PA participation. Our PL model for older adults uses an ecological approach to integrate PL into older adult’s lifestyles. This model integrates all components (intrapersonal, interpersonal, organizational, community, and policy) as being involved and intertwined in the promotion and adoption of PL. Elements within each component support how each might influence PL adoption by the older adult. Understanding the interactions between components and elements which facilitate PL education and practice may ultimately provide a new and effective blueprint to specifically target PA promotion and adherence for all older Canadians.

Future Directions (Action Items):

- Evaluation, refinement, and application of the older adult PL model.
- Improve upon methods to reliably and validly assess key components of the operationalization of PL for older adults, especially at the interpersonal level of the model.
- Application and adaptation of PL models and research studies to vulnerable groups who have low levels of PL (e.g., low income seniors, Aboriginal and other ethnocultural groups, and socially isolated older adults).
- Formative and effectiveness evaluation studies of best practices and innovative interventions to promote PL uptake. Including how the model will influence and be used by frontline people and older adults.
- Integration with other government initiatives aimed at enhancing healthy lifestyles and preventive health behaviours of older adults. Including investigating policy channels that will be most effective in promoting PL.

## Abbreviations

CWG: Collaborative working group
PA: Physical activity
PL: Physical literacy
